# Le fléau de la falsification des vaccins

**DOI:** 10.48327/mtsibulletin.2021/128

**Published:** 2021-09-06

**Authors:** P. Saliou, Q. Duteil, M. Gentilini

**Affiliations:** 1 Organisation PanAfricaine de Lutte pour la Santé (OPALS) 56-58, rue des Morillons 75015 Paris, France

**Keywords:** Vaccination, Falsification, Contrôle qualité, Santé publique, Criminalité, Afrique, Monde, Vaccination, Falsification, Quality control, Public health, Crime, Africa, World

## Abstract

Fake vaccine trafficking is a recent but growing phenomenon, which represents a severe threat to public health. During the Covid-19 pandemic, Covid vaccines have been a prime target for traffickers, but all types of vaccines are falsified by profit-hungry criminals.

The consequences of falsification on global health are serious: decline in vaccination coverage, loss of control of epidemics claiming yet more victims, resurgence of diseases that been under control. Fake vaccines also fuel the mistrust of populations towards science and towards the authorities.

In order to tackle this scourge, a general and coordinated mobilization of all actors concerned is urgently needed: health professionals, political decision-makers, police and customs forces, judges and prosecutors, without forgetting the crucial awareness-raising of public opinion.

## Introduction

Chaque année, dans le monde, 2 à 3 millions de vies au moins sont sauvées grâce à la vaccination [18]: l'une des plus belles réussites de la médecine.

Un siècle après la première « vaccination » contre la variole, basée sur l'observation géniale du médecin anglais Edward Jenner en 1796, Louis Pasteur identifiait les micro-organismes, leur rôle dans la propagation des maladies infectieuses et démontrait le mécanisme scientifique de la vaccination. La formidable épopée de la prévention vaccinale était lancée.

Les succès éclatants obtenus au Nord sont reproduits au Sud, dans les régions les plus défavorisées, avec la mise en place en 1974 du Programme élargi de vaccination (PEV) [24]. En 2020, l'arrivée des vaccins à ARN messager (ARNm) contre la Covid-19, développés en un temps record, ouvre de nouvelles perspectives et autorise de nouveaux espoirs.

Mais la vaccination est attaquée. Non seulement par les groupes « anti-vaccins », mais aussi par des criminels qui, au péril de la santé des populations, falsifient les vaccins comme les médicaments pour leur profit. Loin d’être un phénomène isolé, le trafic de faux vaccins, qui frappe avant tout les pays les plus pauvres, s'amplifie, s’étend sur tous les continents et constitue une grave menace pour la santé mondiale.

## Historique des faux vaccins

Le premier cas documenté de falsification d'un vaccin remonte à 1995. Alors qu'une épidémie de méningite cérébro-spinale à méningocoque du groupe A (MCSm) se répand au Niger, une campagne de vaccination en urgence est entreprise pour casser la courbe épidémique. Prônée dès la fin des années 1970 du fait de la disponibilité d'un vaccin polyosidique spécifique, cette stratégie a déjà fait la preuve de son efficacité [25]. Mais cette fois, le manque d'infléchissement de l’épidémie [6] et l'aspect douteux des vaccins [8] conduisent les équipes de Médecins sans frontières (MSF) et les autorités sanitaires du Niger à alerter le laboratoire producteur. Après vérification des numéros de lots et analyse en laboratoire, la suspicion est confirmée: les vaccins sont des faux, ne contenant aucun principe actif ! La falsification concerne 88 000 doses de vaccin antiméningococcique, en provenance du Nigéria, dont environ 60 000 déjà administrées [19] seraient responsables de la mort d'au moins 3 000 personnes.

Vingt ans plus tard, avec 30 000 cas répertoriés chaque année en dépit d'une baisse significative de l'incidence depuis 2010 grâce à la vaccination systématique, les pays de la « ceinture de la méningite » décrite par Lapeyssonnie paient toujours un lourd tribut à cette infection. Les efforts contre celle-ci sont constamment sabotés par les trafiquants-tueurs: en 2015 [11], 2017 [13] et 2019 [15], des vaccins falsifiés ont été identifiés en Afrique de l'Ouest, en particulier au Niger.

Si la vaccination en réponse immédiate à une épidémie a été propice pour révéler l'existence de la falsification des vaccins, il est très vraisemblable que de faux vaccins circulaient déjà auparavant. En effet, dans les campagnes de vaccination « préventive » à large échelle contre d'autres maladies, un faux vaccin est beaucoup plus compliqué à identifier: dans les pays du Sud, en l'absence de pharmacovigilance et de suivi des personnes vaccinées, si aucun effet indésirable grave ne vient révéler une fraude, comment la repérer ?

Les conséquences sanitaires sont graves à l’échelle individuelle et à l’échelle collective: échec de prévention de la maladie visée par le vaccin, propagation et perte de contrôle de l’épidémie, multiplication des victimes, perte de confiance du public… Leur ampleur les rend difficiles à mesurer avec précision, retardant la prise de conscience de ce fléau.

En 2016, l'Indonésie a ainsi découvert qu'une organisation criminelle s'adonnait au trafic de faux vaccins sur son territoire depuis treize ans [9]: vaccins pédiatriques combinés contre la diphtérie, le tétanos, la poliomyélite, la coqueluche et les infections à *Haemophilus influenzae* b, vaccins contre l'hépatite B et vaccin BCG contre la tuberculose ont été falsifiés et distribués dans neuf provinces et près de quarante cliniques et hôpitaux à travers le pays [27]. Face au scandale, dans l'incapacité d’évaluer le nombre d'enfants ayant reçu ces faux vaccins, le gouvernement indonésien a dû lancer en urgence une campagne de re-vaccination massive [29] et procéder à un profond remaniement de l'agence du médicament qui, alertée trois ans plus tôt de l'existence d'un trafic, n'y avait pas accordé d'attention [23].

Comme pour les médicaments, tous les vaccins peuvent être falsifiés. Ainsi, récemment :

- au Bangladesh, en 2016, falsification du remarquable vaccin contre la fièvre jaune [12], (dont une seule injection confère une protection à vie contre cette maladie potentiellement mortelle);
- en Ouganda, en 2018, falsification des vaccins contre l'hépatite B [10];
- au Bangladesh de nouveau, en 2019, distribution du contenu de 8 000 cartons de faux vaccin anticholérique par voie orale [14];
- aux Philippines, en 2019, les trafiquants ont mis à profit la pénurie de vaccins antirabiques pour diffuser des lots de vaccins humains et de sérum contre la rage falsifiés [16].

Les vaccins vétérinaires sont, eux aussi, concernés: un vaccin falsifié contre la rage a été identifié en Pologne, en 2017 [1], et le trafic est probablement largement répandu en Afrique.

## Les vaccins anti-covid, aujourd'hui

Depuis le début de l'année 2020, la recherche scientifique a permis de concevoir, développer, produire et mettre sur le marché en un temps record des vaccins sûrs et efficaces contre la Covid-19, avec l'espoir d'une maîtrise de la pandémie dans les prochains mois. Mais, prompts à réagir, les criminels ont vu, dans le lancement de cette campagne vaccinale mondiale, une opportunité pour de juteux trafics [4] :

- au Sud, profitant de l'accès difficile à ces vaccins de qualité et des carences des circuits pharmaceutiques, les trafiquants distribuent et administrent les faux vaccins dans des circuits parallèles (cliniques privées, centres de « santé naturelle », entreprises…). Au Mexique [17] et en Equateur, entre autres, des dizaines de milliers de personnes ont reçu une ou plusieurs injections de ces vaccins falsifiés [30].
- au Nord, exploitant les difficultés d'approvisionnement et la confusion engendrée par une communication alarmiste, excessive, souvent négative ou seulement dubitative, les trafiquants diffusent de faux vaccins sur la face cachée (*darkweb*) d'Internet [7] à des prix s’élevant jusqu’à 1 000 dollars la dose [28].

Dans les pays du Nord, en dehors d'Internet, incontrôlable, les procédures extrêmement rigoureuses d'autorisation de mise sur le marché et de contrôle des vaccins [26] (contrôles internes, à tous les stades de la fabrication, par l'industriel, et externes, par l'Agence du médicament qui délivre les certificats de libération des lots) garantissent leur qualité et leur sécurité. Même dans les situations de crise, le circuit est parfaitement surveillé et garantit la distribution de vaccins authentiques et sûrs.

Dans les pays du Sud, en particulier en Afrique, les populations ne bénéficient pas d'une telle protection. Les circuits pharmaceutiques, fragiles et complexes en raison de la multiplicité des fournisseurs et des modalités d'importation (firmes pharmaceutiques, bailleurs internationaux, dons bilatéraux.), sont beaucoup plus vulnérables, surtout dans un contexte d'urgence, et représentent une cible privilégiée pour les trafiquants. Au Mexique, dès janvier 2021, des groupes relevant du « crime organisé » avaient mis sur pied des laboratoires clandestins dans le but de produire et distribuer des vaccins falsifiés contre la Covid-19. Afin d'infiltrer le circuit légal, les cartels planifiaient de voler des cargaisons de vaccins authentiques destinées aux hôpitaux, en vue de récupérer les conditionnements et d'insérer leurs produits falsifiés dans des lots autorisés [5]. En outre, dans de nombreux pays, la corruption rampante ou massive, à tous les niveaux de la société, alimente le trafic de médicaments et vaccins falsifiés, et lui permet de prospérer.

## Que faire?

La vaccination est attaquée sur tous les continents: calomniée et prise à partie par des groupuscules sectaires et complotistes, mise en doute par une opinion publique qui n'a pas connu ou a oublié la gravité des maladies infectieuses (croup, poliomyélite…), usurpée par des truands qui mettent en danger la santé des populations et risquent d'accroître la défiance. Face à cette menace croissante et mortifère, il faut, d'urgence, en appeler à une mobilisation générale.

### Au sud

Les circuits pharmaceutiques doivent être impérativement renforcés, en donnant aux agences de régulation les ressources humaines, financières et techniques ainsi que l'appui politique nécessaires à leur mission de surveillance et de contrôle des produits de santé, notamment des vaccins.

Ce problème n'est pas nouveau. Déjà en 1987, une réunion sur la fabrication et la distribution des produits biologiques humains et vétérinaires, organisée à Dakar par l'ONUDI [22], avait eu pour objectif de discuter du meilleur moyen de développer des unités de contrôle de qualité pour les produits biologiques, en particulier pour les vaccins, non seulement pour les pays possédant des unités de fabrication mais aussi pour les pays importateurs. Deux laboratoires régionaux de contrôle des vaccins vétérinaires avaient été mis en place (à Dakar et Addis Abeba), mais n’étaient pas opérationnels [21].

Un quart de siècle plus tard, les deux laboratoires vétérinaires ont fusionné, sous l’égide de l'Union africaine, en une structure unique: le Centre panafricain des vaccins vétérinaires (PANVAC), reconnu centre de référence pour le contrôle de la qualité des vaccins par la FAO [3, 31].

Concernant les médicaments et vaccins humains, en revanche, tout reste à faire: aucun laboratoire en Afrique francophone n'est en mesure de contrôler des vaccins; aucun ne bénéficie de la pré-qualification de l'Organisation Mondiale de la Santé [20].

### Au nord

Le contrôle des vaccins avant leur libération sur le marché est assuré par des laboratoires nationaux indépendants des producteurs. La société civile doit être sensibilisée aux risques des vaccins falsifiés en refusant toute proposition émanant d'Internet (« les vaccins, comme les médicaments, ne sont pas des marchandises comme les autres ! »).

### Partout

L'engagement des autorités politiques de santé avec les forces de police, des douanes, les juges et les procureurs, doit être décidé et doté de moyens pour identifier plus rapidement les fraudes et poursuivre leurs auteurs en criminalisant les actes, grâce à la Convention Médicrime du Conseil de l'Europe [2].

Le combat doit aussi porter sur l'opinion publique, par une information rigoureuse permanente et une lutte sévère contre la désinformation. Les professionnels de santé, en première ligne, doivent être sensibilisés à ce trafic et à ses conséquences désastreuses. Leur attitude doit être exemplaire en se faisant vacciner, en consolidant leurs connaissances, en affûtant leurs arguments pour convaincre leurs patients et répondre à leurs interrogations, en s'opposant catégoriquement aux discours « anti-vaccins ».

## Conclusion

Face à la pandémie de Covid-19, l'humanité se tourne à nouveau vers la vaccination. Grâce à une exceptionnelle mobilisation scientifique mondiale, un an seulement après l’émergence de la maladie, la sortie de crise est en vue. Mais le mal plus insidieux du trafic de vaccins falsifiés, lui, perdurera. Il doit donc être combattu, à tous les niveaux, avec autant de vigueur que la Covid-19.

## Conflits D'intérêts

Les auteurs ne déclarent aucun conflit d'intérêt.
